# Needs and views on eye health and women’s empowerment and theory of change map: implication on the development of a women-targeted eyecare programme for older Zanzibari craftswomen

**DOI:** 10.1136/bmjophth-2023-001292

**Published:** 2024-02-23

**Authors:** Fatma Omar, Kayleigh McCluskey, Eden Mashayo, Ai Chee Yong, Damaris Mulewa, Christine Graham, Carlos Price-Sanchez, Omar Othman, Ronnie Graham, Ving Fai Chan

**Affiliations:** 1 Zanzibar Ministry of Health, Zanzibar, Tanzania; 2 Centre for Public Health, Queen's University Belfast, Belfast, UK; 3 Vision Care Foundation, Dar-es-Salaam, Tanzania; 4 Independent Researcher, Nairobi, Kenya; 5 Vision Aid Overseas, London, UK; 6 University of KwaZulu-Natal College of Health Sciences, Durban, South Africa

**Keywords:** Public health, Vision

## Abstract

**Objectives:**

To assess needs and views regarding eye health and empowerment from craftswomen’s perspectives to develop a theory of change (ToC) for a women-targeted eyecare programme.

**Material and methods:**

Eighteen stakeholders participated in a 2-day consultation workshop in Zanzibar. The composition was (1) 15 women and 3 men; (2) Unguja (n=8), Pemba (n=6) and Tanzania mainland (n=4) and (3) craftswomen (n=14) and governmental stakeholders (n=4). Thematic analysis determined the craftswomen’s needs and views regarding eye health and empowerment and subsequently inputs, activities, outputs, outcomes and impact to develop the programme’s initial ToC. In refining the initial ToC, we used insights from a qualitative study suggesting that improved near vision is perceived by craftswomen as a potential source of empowerment across economic, psychological, social, political and educational dimensions.

**Results:**

The eye conditions experienced by the craftswomen were eye irritation caused by foreign bodies, the need for near spectacles and other eye morbidities. They were advised by the cooperatives to visit eye health centres for treatment. The main barriers to accessing services were inaccessibility and unaffordability of eye services and a lack of eye health knowledge and practices. Nineteen subthemes on women empowerment (economic n=4, social n=4, psychological n=6, education n=2 and political n=3) were obtained. We created a ToC on how investing in improving craftswomen near vision could achieve empowerment.

**Conclusion:**

The participants provided insights into their needs and how they would like the eyecare programme to be implemented and how they see they could be empowered in the process.

**WHAT IS ALREADY KNOWN ON THIS TOPIC**

- The vulnerable position, high prevalence of presbyopia and low correction rate among older Zanzibari women called for a women-targeted eye care programme.
- Older craftswomen could provide critical information that could help in planning, implementing and evaluating an eyecare programme that aims to correct their vision and empower their lives.

**WHAT THIS STUDY ADDS**

- Not only did the women report their needs and current eyecare situations among the craftswomen, but they also helped us to understand how they would like the eyecare programme to be implemented and how they see they could be empowered in the process.

**HOW THIS STUDY MIGHT AFFECT RESEARCH, PRACTICE OR POLICY**

- The documentation of the process could assist programme implementers and policy-makers in systematically developing a women-targeted eyecare programme using a participatory approach.

## Introduction

Presbyopia is an age-related near vision impairment most commonly affects people 40 years and older.^1^ If left uncorrected, presbyopia can cause visual discomfort and reduce one’s ability to perform near-visual tasks.^2^ It is the most common cause of vision impairment, affecting 1.1 billion people worldwide.^1^ However, presbyopia has been shown to disproportionally affect low-income and middle-income countries (LMICs). The World Report on Vision also reported that African people display a younger onset of presbyopia and more severe symptoms than those in Europe and North America.^1^ Although presbyopia can be treated inexpensively with spectacles, correction rates in LMICs are extremely low—for example, 6% in Tanzania^3^ and 7% in rural South Africa.^4^ High rates of untreated presbyopia can cause large-scale economic strain, with estimated global productivity losses due to presbyopia exceeding US$25 billion in productive years.^5^

Unfortunately, not a single LMIC is on track to achieve Sustainable Development Goal 5 (gender equality) by 2030.^6^ Women in LMICs are over-represented in informal and vulnerable employment—7.8% higher than men.^7^ Regarding eye health, the Lancet Commission reported that women, and older women most severely, disproportionately lack access to eyecare services compared with men.^6^ However, case studies in Vietnam,^8^ and studies in South Africa^9 10^ and India^11^ have shown that providing comprehensive eye health interventions to women in settings such as the workplace can be a low-cost, sustainable and practical approach to reducing the prevalence of presbyopia and improving work productivity.

Zanzibar is an autonomous region of the United Republic of Tanzania. It consists of the two main islands of Unguja and Pemba. Though the Islands’ populations are almost entirely Muslim, their cultural practices are distinctive. In 2019, about 51% of people in Zanzibar were women, 23% of whom were the head of their households.^12^ These women supported an average of nine unemployed persons, while men supported only four.^12^ Furthermore, 44% of women heads in households had no education. Consequently, many participated in the home industry as craftswomen to generate income—economic activities heavily dependent on near vision.^12^ The prevalence of presbyopia in 2010 in Zanzibar was high (89.2%), but spectacle coverage was relatively low (17.6%).^13^ The same study shows that spectacles’ correction can significantly improve quality of life scores (effect size=1.15–3.90).^13^ Craftswomen who cannot produce quality goods due to their poor near vision will likely earn lower incomes than they might otherwise obtain. Their resulting economic vulnerability, lower levels of education on average and unequal social status all may contribute to diminished access to health services, low confidence and inability to claim one’s rights (disempowerment).^14 15^

Empowerment is ‘the process of enhancing an individual’s or group’s capacity to make purposive choices and to transform those choices into desired actions and outcomes’.^16^ It is to allow women to have a sense of autonomy over their lives. VeneKlasen and Miller^17^ called this an expression of power. Further, Lombardini *et al*^18^ recognise that empowerment could occur at three levels: individual, relational or environmental. Mandal’s^19^ further grouped empowerment into five categories: social, psychological, educational, economic and political.

Recognising the need to realise women’s full human rights, the Revolutionary Government of Zanzibar has committed to attaining gender equity, equality and empowerment among Zanzibari women. Through the Zanzibar Women’s Cooperative, craftswomen engage in income-generating crafts activities (embroidery, sewing clothes and making solar lamps). However, most craftswomen aged 35 years and older had uncorrected presbyopia.^20^ These findings strongly indicate that gender-sensitive eye health programmes are needed. Currently, there is only one privately owned vision centre on each of the bigger islands of Unguja and Pemba. Optometry services such as primary eye care, refraction and spectacle dispensing are provided at these vision centres. Higher ophthalmological care is provided at the tertiary hospital on Unguja Island. These vision centres and eye hospitals are situated in densely populated areas, where women’s cooperatives are situated within a 5–20 km radius. Unfortunately, poor transportation systems and infrastructure meant services were not easily accessible to women. A willingness-to-pay study in 2010 also indicated that the cost to access services was high where public transport costs from US$1 to US$2 depending on distance while the cost of a pair of ready-made glasses sold at the public hospital was about US$3.90 while the local private practices charges about US$12.00–US$14.00.^21^ Hence, the Ministry of Health, Social Welfare, Elderly, Gender and Children Zanzibar and the Queen’s University of Belfast collaborated in 2022 to deliver eye care service to the craftswomen and thus, the Women’s Empowerment through Investing in Craftswomen’s Eyesight (WE-ZACE) pilot programme^22^ was conceived.

To our knowledge, WE-ZACE is the first women-targeted eye care programme in sub-Saharan Africa. No published literature documented the development of such an eye care programme. To help effective planning, implementation and evaluation of the WE-ZACE, a consultation workshop was conducted to answer two main questions: (1) What are the needs and views regarding women’s eye health from the craftswomen’s understanding? and (2) What is the potential impact of the WE-ZACE programme on the empowerment of craftswomen in Zanzibar?. With information from the workshop, we identified the inputs, activities, outputs, outcomes and impact needed to develop the programme’s theory of change (ToC) map. A ToC is a participatory theory-driven approach to programme design and assessment,^23^ which could assist the understanding of how and why the WE-ZACE programme works. The WE-ZACE ToC has two primary programme design purposes: (1) to foster a shared understanding among stakeholders and implementers on the expected empowerment WE-ZACE can bring to the craftswomen and (2) to explain the pathway to achieve the impact.

## Material and methods

### Patient and public involvement

This workshop, conducted from 24 October 2022 to 25 October 2022, is part of the WE-ZACE patient and public involvement strategy. As described in this article, the public and the local key stakeholders were invited to attend the consultation workshop and participate in refining the ToC map.

### Sample and sampling

The participants were decided based on an initial list of approximately 60 stakeholders identified by the research team. The steering group then selected 18 information-rich key stakeholders to represent those involved in the WE-ZACE programme design, implementation and beneficiaries. The participants were considered information-rich as they were women who had worked in the crafts for more than 5 years, covered three main types of crafts (tailoring, beading/weaving and pottery), who were in leadership positions and understood the local craft industry and cultural dynamics well. The participants were chosen based on (1) sex: women (n=15) and men (n=3); (2) roles: craftswomen (n=14) and governmental stakeholders (n=4) and (3) location: Unguja (n=8), Pemba (n=6) and Tanzania mainland (n=4). Craftswomen from Unguja and Pemba islands were both chosen because of the difference in socioeconomic status, with Pemba having a higher poverty rate than Unguja island. Tanzanian mainland participants were women entrepreneurs who were actively involved in women’s empowerment advocacy efforts.

Participants were split into three heterogeneous groups, each with six participants, to allow for active discussion and participation (table 1).

**Table 1:** Description of workshop participants in each group

	Group 1	Group 2	Group 3
Sex			
Male	1	1	1
Female	5	5	5
Age (years)			
35–45	2	1	4
46–55	3	2	1
>55	1	3	1
Location			
Pemba	4	0	2
Unguja	2	5	1
Tanzania	0	1	3
Organisation			
Women cooperatives	5	5	4
Government	1	1	2
Total	6	6	6

### Procedure

The first part of the discussion topics aimed to understand the needs, examine intervention components and consider implementation barriers and facilitators of the WE-ZACE programme. To understand women’s empowerment among the craftswomen, Mandal’s concept and types of women’s empowerment were used to guide our fundamental building blocks of women’s empowerment (ie, economic, psychological, social, education and political) because these empowerment elements are relevant to our unique sample of women entrepreneurs.^19^ Two main questions were asked: (1) What is your understanding of women’s empowerment (or in Swahili, a strong woman or woman who can take control of their lives and make decisions) in Zanzibar?, with probing questions exploring the five building blocks of empowerment stated by Mandal and (2) How do you think better vision can help empower craftswomen?.

Before the workshop, FO and OO telephonically contacted the women’s cooperative managers and craftswomen and explained the facilitators’ role and background and the workshop’s aim. Two trained Swahili-speaking facilitators (the national eye care coordinator with, FO and a public health optometrist, EM) conducted the 2-day workshop to allow for full and rich discussion. Each session included a 30 min discussion, followed by a 30 min break for the write-up and reflection and an hour for feedback.

A set of deductive codes on eye problems, interventions, barriers, facilitators and types of empowerment guided the analysis of the workshop notes.^24^ The workshop notes were recorded in Swahili to ensure accuracy. Our bilingual facilitator (FO and OO) translated them into English and back-translated them by our Swahili-speaking analyst (DM). Two analysts (KM and DM) worked independently; all the workshop notes were read first to familiarise the analysts with the data. Then, they listed and grouped them into preliminary themes and discussed them with the senior author (VFC). For example, we wanted to ascertain the types of eye problems the craftswomen were experiencing at their workplace. We, therefore, labelled ‘Eye problems’ as a priori categories and coded (1) reaction to foreign bodies, (2) suspected need for spectacles and (3) eye conditions that require ophthalmological care. Once these categories were established, the responses were arranged according to their salience. We annotated all the meeting notes using their group number (groups 1, 2 and 3) to group codes that shared commonalities. After conducting individual analysis, KM and DM compared and discussed their findings. Any disparities were addressed with the help of VFC, as required, to arrive at a final classification.

During the workshop, FO and EM developed an initial ToC that depicted how investing in improving older craftswomen’s near vision could lead to women’s empowerment. Subsequently after the workshop, by using the qualitative data from the workshop, KM and DM produced independent ToCs comprising interconnected inputs, activities, outputs and outcomes, leading to a final impact alongside a set of theorised determinants to be influenced. Then, the results were compared, discussed and revised with VFC, FO, EM OO and five selected craftswomen who attended the workshop (12 November 2022–18 November 2022). A provisional ToC was then constructed. Subsequently, a qualitative study was conducted with 24 craftswomen (7 April 2022–21 April 2022) to understand how they perceive the relationship between spectacle correction and empowerment where the methodology and findings have been discussed in detailed in a separate publication.^25^ In summary, the qualitative findings showed that the craftswomen perceived presbyopic correction to be a catalyst to achieve economic, social, psychological, education and political empowerment.^25^ These findings were used to produce the revised ToC with five selected craftswomen who attended the workshop (12 November 2022–18 November 2022).

## Results

### Common eye conditions found among the craftswomen

The respondents mentioned that the three most common eye conditions found among the craftswomen were (1) reaction to foreign bodies [‘*we saw women experiencing itchy eyes, tearing and painful eyes from the dust and debris.’ Group 2*], (2) need for spectacles [‘*could not see the details and prints clearly when they are too small.’ Group 2; It is very difficult to cut patterns… and sew clothes because we cannot see clearly, especially when the lines are fine and at close distance.’ Group 3*] and (3) eye conditions that require ophthalmological care [‘*we heard a few women had become blind from white eyes’ Group 1; ‘…also high eye pressure. Group 2’*]. When experiencing symptoms of these conditions, the craftswomen were often advised by the cooperative managers to ‘*visit the nearest health centre or eye clinic’* (Group 2) and ‘*to be examined and receive treatment from doctors and advice on eye care’* (Group 3).

### Barriers to eye health services uptake and solutions suggested by craftswomen to resolve their eye health issues

Lack of access to eye care services [‘*Long distances to eye clinics. It takes us one whole day to travel to the eye clinic in MM to get our eyes tested.’ Group 2*], and financial barriers [‘The *spectacles and eye medicine are so expensive. Who can afford them?’ Group 3*] have been highlighted as barriers to service uptake. The craftswomen proposed that eye health treatments such as spectacles, medication, eye examination and diet could be provided to them at the cooperatives or for free. These solutions were based on their observations where many peers faced difficulties when producing their crafts *[‘Just like me, many of the women working at the groups, have problems looking at the (needles of sewing] machines. One or two of them who wear glasses seemed to have no problems with that. I think glasses could help us.’ Group 2; ‘You know Madam A, her eyes (are) always red. One time, she rubbed her eyes so hard, there was blood in her eye. I tell you, the dust is bad. She put in some eyedrops from the medicine shop and seemed to work. We should stock those medicines in our factory.’ Group 1*]. Some craftswomen were aware that there is a need for eye examination when they become older *[‘women, especially us women! Our body is not as good as when we are young… including our eyes. The doctors should help provide us the eye examinations every year.’ Group 1.’*] and that a good diet plays a role in protecting their vision [‘*I was told carrots and sweet potatoes are good to protect our eyes.’ Group 3*].

Another obstacle to accessing eye health services in the local community was poor eye health knowledge and practices in the community [‘*You know, many of the women are ignorant on health care. They do not know it’s important to have good eyesight. I must say, it’s only until now that you are here to show us glasses could help us, many would not have known! Also, there is fear in the community for eye treatment.’ Group 2*]. They also have prevailing reservations about the usage of Western medicine and spectacles [‘*If we have a problem, we go to our healers. They give us herbal medicines. Medicines from hospitals have chemicals and the traditional medicines do not have chemicals.’ Group 3*; ‘*Some fear that wearing spectacles makes your vision poorer. For example, I do not want my kids to wear them all the time too.’ Group 1*]. Hence, eye health education was highlighted unanimously as an important way to address this issue [‘*the only way we can overcome this issue is to educate people on eye health problems in the women groups.’ Group 1]*. To make sure the eye health issues are dealt with effectively, women themselves should be equipped to identify peers with eye problems [‘*to build capacity of the entrepreneurs to be able to look for people with [eye] problems at the cooperative’ Groups 1 and 3*].

As a prevention method from eye-related hazards, the craftswomen also proposed improvement in their workplace safety by having equipment safety goggles to protect them from chemicals used when producing their crafts [‘*to be facilitated with modern business equipment to protect ourselves from chemicals.’ Group 1*].

### Craftswomen’s understanding of the characteristics of empowered women

When we further probed their understanding of the characteristics of empowered women, 19 subthemes on the 5 women empowerment domains were obtained (economic, social, psychological, education and political) (table 2).

**Table 2:** Craftswomen’s understanding of the characteristics of empowered women

Theme	Subthemes	Illustrative quotes
Psychological	To believe in yourself Understand areas that one is good at To have peaceful thoughts To have a wake-up call (self-awareness) To be daring To be able to do a self-assessment	‘…is one who believes in themselves and can speak her mind.’ Group 3 ‘…is one who can ask themselves, how can I do better?’ Group 2 ‘…be calm but brave. We face a lot of challenges at home and work. But, Insha’Allah, we can overcome them. [Clapping in the room].’ Group 2
Economic	To improve the financial situation To be able to employ people around you To achieve set business goals	‘…we need to make more money from our business. There are too many challenges stopping us. We cannot sell our products outside of Zanzibar. Not even to Tanzania! The market is restricted. [agreed unanimously by participants].’ Group 1 ‘…as a successful businesswoman, we must be able to recruit people around us to produce our products faster and better. Especially the younger people.’ Group 1 ‘…to be empowered as a businesswoman, we can set goals. Not everyone knows how to do it now, madam.’ Group 3
Political	To be able to provide advice in different situations Vying for leadership positions to elect and be elected To be able to listen and follow government guidelines on entrepreneurship	‘…we should be strong enough to be in leadership positions.’ Group 1 ‘We can advise the community, cooperatives and our peers… also our family issues.’ Group 2
Social	Capacity to eradicate poverty and sexual harassment Identify sexual harassment victims and co-parenting To be able to support other craftswomen To eradicate dependency	‘…again, I have to say, we face a lot of challenges. We know some women were harassed by other men. As a strong woman, we must know how to fight back.’ Group 2 ‘Yes. Some women suffered quietly, we must know who they are and help them.’ Group 3 ‘No need to rely on others’ Group 3 ‘…we can have the power to not be poor… and support other women in our groups.’ Group 1
Educational	Educating the community to better understand themselves Educated on entrepreneurship	‘Once we have learned the skills we need, we can teach the community and our peers how to become better in their work and lives.’ Group 2

**Figure 1:**
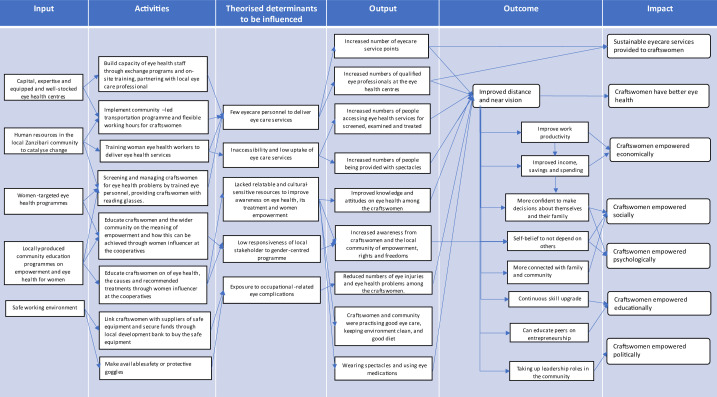
Visualisation of key elements of the theory of change map for the WE-ZACE programme. WE-ZACE, Ministry of Health, Social Welfare, Elderly, Gender and Children Zanzibar.

Subsequently, the initial ToC for the WE-ZACE pilot programme was developed (online supplemental figure). The initial ToC was then revised based on the findings of a subsequent qualitative study^25^ to produce a final ToC (figure 1). It outlines that investment is needed in stocking up on eye health treatment, training human resources for eye health, implementing women-targeted programmes, developing eye health education and ensuring a safe working environment. Craftswomen also wished to be able to market their products to mainland Tanzania and outside of Tanzania to increase the sales of goods and maximise earnings. In return, they will have positive health and eye health practices and improved productivity, income, business opportunities, confidence and family and political engagement. The impact WE-ZACE attempts to achieve is that craftswomen are empowered politically, socially, economically, psychologically and educationally.

10.1136/bmjophth-2023-001292.supp1 Supplementary data

## Discussion

The WE-ZACE consultative workshop was conducted to obtain input from key local stakeholders. We found that the main eye-related challenges among many craftswomen were eye irritation from foreign bodies and the need for spectacles. They were most frequently asked by the cooperative managers to visit eye health centres for examination and treatment if suspected to have eye problems. However, inaccessibility to affordable services, as well as poor eye health knowledge and practices in the community, served as impediments to the utilisation of the service.

Eye irritation caused by foreign bodies is common in a dusty working environment.^26^ Vasu *et al* observed that eye irritation might decrease work productivity and inferior product quality, reducing earnings.^26^ The same study also found that in LMICs, occupational eye injury can be severe and contributes significantly to work absences and loss of productivity as the workers are exposed to foreign bodies such as dust and wood shavings produced via agriculture, carpentry, chiselling and hammering activities.^26^ Another study by Reddy *et al* showed that inaccessibility to spectacles was the main issue affecting rural workers in India, which our study agrees with.^11^ These findings warrant the need for a health and safety policy and the provision of protective goggles and spectacles to address the issues of eye-related occupational hazards and good vision. Subsequently, a survey among craftswomen in Zanzibar found that the prevalence a high proportion of older craftswomen (29.7%) had vision impairment.^27^ More than two-thirds needed either distance or near corrective spectacles and the rest needed further ophthalmological care due to posterior eye diseases.^27^

Two key barriers towards uptake of eyecare services were lack of access to affordable services, and poor eye health knowledge and practices in the community. Traditional medicine is intrinsically linked to culture, society and healthcare in Tanzania,^28^ as was also shown by the responses from the participants. This treatment option is often preferred over conventional therapies due to accessibility, availability, affordability, cultural acceptance, and spiritual, religious, and sociological values, as shown in Ghana.^29^ A study from rural Malawi found that despite 76.8% of participants believing treatment from an eye health centre would be the least expensive option, 72% opted for traditional medicines.^30^ Given that traditional medicine plays a critical role in people’s lives, integration of traditional medicine into primary eye care, coupled with close support and monitoring of its contribution, may reduce poor outcomes due to delayed treatment.

Although not reported by our participants, gender inequality might contribute to limiting craftswomen’s access to eye care services. It is common to see fewer women than men utilising necessary eye health services due to the prioritisation of men’s health in struggling economic climates and the perceived social superiority of men.^30^ In our study, despite the craftswomen being aware of the eye health problems faced and were given referral advice accurately, the main reason craftswomen did not access eye health services might be due to women are often less likely to have access to financial resources than men, making it difficult for them to afford eyecare services and that some traditional gender roles in Zanzibar may discourage women from seeking eyecare, or may make it difficult for them to travel to eyecare providers.

Our findings, and others,^30 31^ indicate the need to identify women with eye problems and increase women’s trust in treatments received at eye care facilities. Previous intersectional analysis among older adults in the Indian Sundarbans showed that one-to-one eye-health education sessions and identification and counselling of people with vision problems by trained female community workers increased eye health knowledge to more than 90% over baseline and doubled the service uptake.^32^ The study also highlighted that using female healthcare workers may have maximised the impact of the eye health education strategy.^32^ In the Zanzibar context, these gender-transformative health strategies have great potential as there is projected to be rapid growth in health employment for women because increasing women’s representation in the healthcare workforce has an economic impact.^33^

The craftswomen also provided their views on the characteristics of empowered women, which were well-aligned to Mandal’s five elements of women empowerment. One interesting observation from the workshop is that craftswomen repeatedly mentioned psychological and economic empowerment. Self-belief and innovation, self-control over one’s work, and an active engagement with one’s role can effectively stimulate enthusiasm for work and improve job performance.^34^ This increased engagement and performance might also have spill-over effects, motivating other team members and leaders.^34^ Bano *et al*’s study on the impact of craftwork on women’s empowerment concurred that psychological empowerment, such as self-confidence, ability to stand up to abuse, increased freedom and self-esteem is of utmost importance to women.^35^ However, this study argues that economic empowerment must first be achieved to attain psychological empowerment. Furthermore, the work of Rowlands^36^ found that empowerment is a dynamic process, starting with the personal and psychological dimensions, which can lead to economic empowerment and eventually influence society and politics. Hence, the complex and dynamic relationship between the different aspects of empowerment requires further research, especially among craftswomen.

Conducting a follow-up qualitative study with the craftswomen, exploring their perspectives on how spectacle correction could empower them, not only provided deeper insights but also validated assumptions raised during the ToC workshop. This effort has significantly enhanced and refined the initial WE-ZACE ToC. The insights gained from this study underscore the critical role of the ToC in guiding implementation, facilitating outcome measurement and evaluating the overall success of the programme. The clear descriptions of the inputs, activities and short-term and long-term outcomes can help clarify goals and communicate the basics of how our initiative works for others.^37^ Vogel’s study supports our use of a ToC, as it demonstrates a real understanding of the thoughts and views of the local community and, in doing so, can make a real difference to people, especially in LMICs.^38^ A ToC can also be mimicked in other communities and environments, allowing other interventions to take stock of their local context and adapt the ToC as needed. We recommend that the ToC be continually monitored and updated when new data is available, periodically evaluated and changes made when necessary.

We emphasise our acknowledgement that our findings act as an initial step in the planning process. Subsequent to the workshop, a programme committee was established. A survey was then carried out to assess the eye health needs and attitudes towards eye health among elderly craftswomen.^27^ The survey revealed a significant prevalence of uncorrected presbyopia (86.6%), accompanied by remarkably low spectacle coverage (0.99%).^27^ These findings establish a baseline for assessing the impact of spectacle correction. Additionally, the same study indicated a positive attitude among craftswomen towards wearing spectacles.^27^

At the first implementation stage, follow-up discussions were held with the Ministry of Health to include presbyopia correction as part of the National Insurance Health scheme, provide human resources to train local women to conduct eye health screening and ensured sufficient spectacles were stocked at the vision centres for future procurement by the craftswomen. Local arts group were also engaged to develop an eye health promotion strategy targeted at women in general.

We conducted eye examinations and offered free eye health services, along with near vision spectacles, to 217 craftswomen experiencing presbyopia.^39^ This initiative aimed to assess their immediate responses in terms of self-rated subjective well-being and work productivity 30 min and 1 hour after correction. Additionally, we conducted interviews with a subset of 20 craftswomen to gain insights into how poor vision impacted their lives.^39^ It showed that the craftswomen had a significant subjective well-being score improvement (2.67; 95% CI 2.49 to 2.85) and self-rated current work performance score improvement (0.85; 95% CI 0.66 to 1.04) shortly after the correction.^39^ The 217 craftswomen provided with near vision spectacles will be followed up for 6 months to understand whether the spectacle correction improved their empowerment. These findings will allow us to revisit and revise the ToC to make sure it reflects the current context of the WE-ZACE programme.

Furthermore, a group of woman business graduates were engaged to assist craftswomen in developing sustainable business plans because access to the trading market is important in achieving empowerment among craftswomen. In the context of Zanzibar, craftswomen wished to be able to market their products to mainland Tanzania and outside of Tanzania. Greater access to trading markets can increase the sales of goods, maximise earnings and improve one’s standing in society. A study among women farmers in rural Africa found that farmers can negotiate better deals for their goods by placing them in markets with high product demands by increasing access to markets.^40 41^ One advantage of the Zanzibari craftswomen is that they function through the women cooperatives that facilitate financial support from mainstream financial institutions. Working through women’s cooperatives has additional indirect benefits, such as developing a culture of saving and getting familiar with the loan system.^40 41^ In our context, providing craftswomen with easier routes to market through consultation with local business leaders and politicians and educating them on loan schemes and negotiation strategies may be beneficial. All these supplementary interventions are designed to enhance the impact of the vision intervention, ultimately working towards empowerment.

The workshop’s greatest strength is using diverse participants, including craftswomen, men, governmental implementers and eye care personnel. Such inclusion and diversity allowed a more representative view of eye health problems and women’s empowerment. Experienced female and male facilitators fluent in Swahili and aware of the local customs and traditions encouraged the participants to express genuine opinions. We acknowledge that limitations exist. The COVID-19 pandemic has restricted robust research fieldwork to observe the dynamics among craftswomen in their real-life environment.

The WE-ZACE project aimed to empower craftswomen by improving their eyesight, and the workshop has helped us understand the local needs. Empowerment through vision starts with better eye health education and broader treatment access. The developed ToC map can guide the design, implementation and evaluation of the WE-ZACE programme.

## Data Availability

Data are available in a public, open access repository. The data underlying the results presented in the study are available from the Queen’s University Belfast Data Repository (DOI: 10.17034/8c58398e-1383-4436-9b6f-4b84db36f34b).
