# Supplementary material for: Needs and views on eye health and women’s empowerment and theory of change map: implication on the development of a women-targeted eyecare programme for older Zanzibari craftswomen
Source: BMJ Open Ophthalmol. 2024 Feb 23;9(1):e001292. doi: 10.1136/bmjophth-2023-001292 (PMC10895232; doi:10.1136/bmjophth-2023-001292)
Supplement: Supplementary data [file bmjophth-2023-001292supp001.pdf]

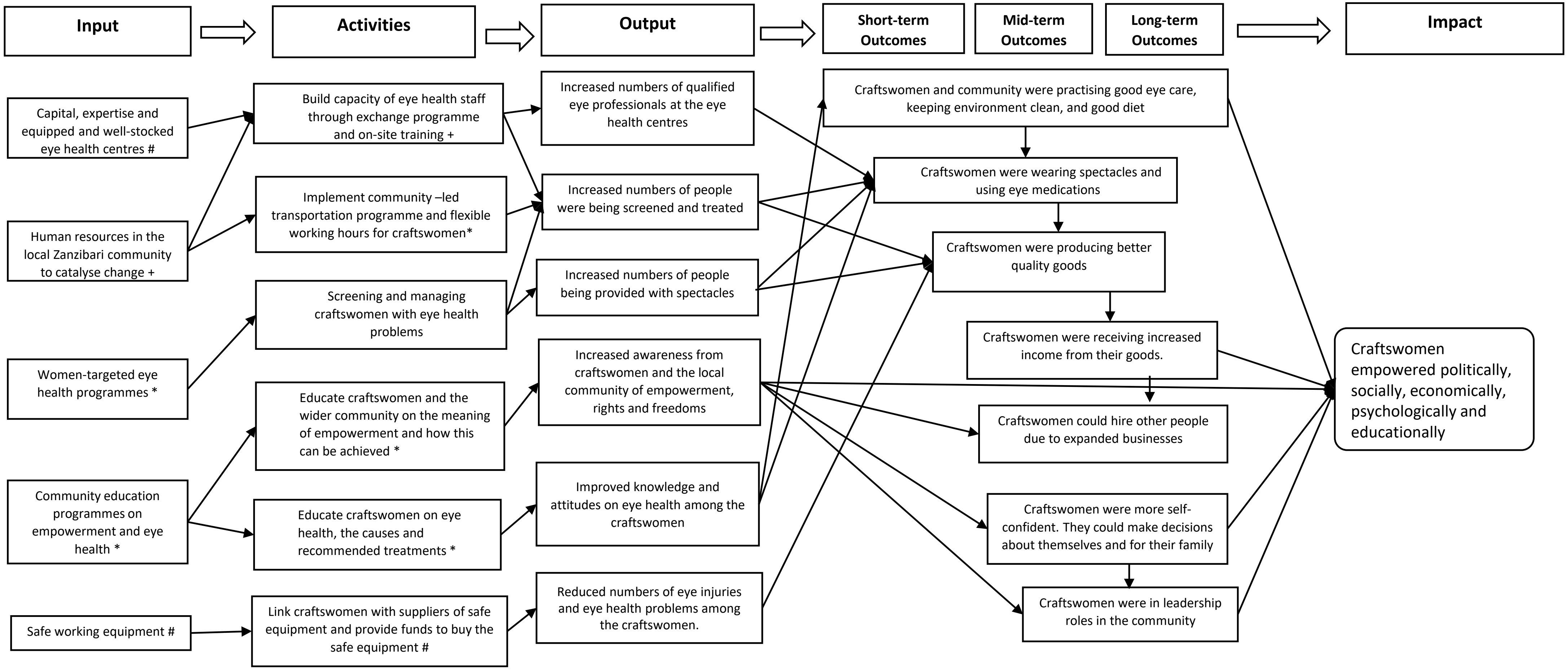

Assumptions

*Target group of craftswomen along with medical professionals, teachers, business leaders, and political leaders in the local community will respond to outreach and engage with the project.

#Adequate resources are in place, supported with internal and/or external funds. Sufficient eye health facilities are equipped with professionally trained personnel and equipment.

+Required numbers of facilitators providing education on eye health and empowerment have sufficient knowledge on the topics and can share this knowledge in a way that the craftswomen can understand.

Supplementary Figure: The initial Theory of Change for the Women’s Empowerment through Investing in Zanzibari Craftswomen’s Eyesight Programme
